# Metabolite Pattern Derived from *Lactiplantibacillus plantarum*—Fermented Rye Foods and In Vitro Gut Fermentation Synergistically Inhibits Bacterial Growth

**DOI:** 10.1002/mnfr.202101096

**Published:** 2022-08-19

**Authors:** Ville M. Koistinen, Maria Hedberg, Lin Shi, Anders Johansson, Otto Savolainen, Marko Lehtonen, Anna‐Marja Aura, Kati Hanhineva, Rikard Landberg

**Affiliations:** ^1^ Institute of Public Health and Clinical Nutrition University of Eastern Finland Kuopio 70211 Finland; ^2^ Food Chemistry and Food Development Unit, Department of Biochemistry University of Turku Turku Turku 20014 Finland; ^3^ Afekta Technologies Ltd. Kuopio 70210 Finland; ^4^ Department of Odontology/Oral Microbiology Umeå University Umeå 90187 Sweden; ^5^ Division of Food and Nutrition Science, Department of Biology and Biological Engineering Chalmers University of Technology Gothenburg 41296 Sweden; ^6^ College of Food Engineering and Nutritional Science Shaanxi Normal University Xi'an 710119 China; ^7^ School of Pharmacy University of Eastern Finland Kuopio 70211 Finland; ^8^ VTT Technical Research Centre of Finland Ltd. Espoo 02044 Finland

**Keywords:** fermentation, lactobacilli, metabolites, microbiota, rye

## Abstract

**Scope:**

Fermentation improves many food characteristics using microbes, such as lactic acid bacteria (LAB). Recent studies suggest fermentation may also enhance the health properties, but mechanistic evidence is lacking. The study aims to identify a metabolite pattern reproducibly produced during sourdough and in vitro colonic fermentation of various whole‐grain rye products and how it affects the growth of bacterial species of potential importance to health and disease.

**Methods and results:**

The study uses *Lactiplantibacillus plantarum* DSMZ 13890 strain, previously shown to favor rye as its substrate. Using LC‐MS metabolomics, the study finds seven microbial metabolites commonly produced during the fermentations, including dihydroferulic acid, dihydrocaffeic acid, and five amino acid metabolites, and stronger inhibition is achieved when exposing the bacteria to a mixture of the metabolites in vitro compared to individual compound exposures.

**Conclusion:**

The study suggests that metabolites produced by LAB may synergistically modulate the local microbial ecology, such as in the gut. This could provide new hypotheses on how fermented foods influence human health via diet–microbiota interactions.

## Graphical abstract

We found seven microbial metabolites being repeatedly produced in various fermentation experiments with strains of lactic acid bacteria as well as during colonic fermentation in vitro. The metabolites also synergistically inhibit the growth of several potential pathogenic bacteria. The diet–microbiota interaction observed may explain some of the beneficial local effects after the consumption of whole grains, particularly rye.

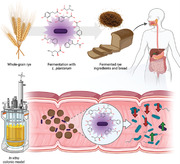

## 1 Introduction

Fermented foods have remained a staple of the human diet for centuries and are an increasingly popular food category.^[^ ^1^ ^]^ Fermentation is a process used to improve the shelf‐life, taste, appearance, and nutrient profile of foods. Beyond that, it may also improve the properties of relevance to human health, for example, through the formation of certain metabolites, but firm evidence of effects in vivo and the underlying mechanism is lacking.^[^ ^2^ ^]^ Lactic acid bacteria (LAB) are beneficial colonizers of both humans and their foods, used in the fermentation of dairy, cereals, vegetables, meat, and other products. They are characterized as Gram‐positive, non‐spore‐forming, anaerobic, and fermentative bacteria. *Lactobacillus* is the largest LAB group, containing at least 261 species and reclassified recently into 25 genera.^[^ ^3^ ^]^ At least three species belonging to this group exist both in the human gut (although their abundance in the gut is generally low) and in fermented foods; *Lactiplantibacillus plantarum* (previously known as *Lactobacillus plantarum*) is one of the most important bacteria in the fermentation of plant foods.^[^ ^4^, ^5^ ^]^ Some strains of *L. plantarum* are also used as probiotics due to their survival of the gastric transit and adherence to gastrointestinal cells,^[^ ^6^, ^7^ ^]^ which makes the species particularly interesting in terms of potential health effects.

Rye (*Secale cereale* L.) has received attention in nutritional science because unlike wheat, it is mainly consumed as whole‐grain products, which are associated with several health benefits, including reduced risk of several chronic diseases.^[^ ^8^ ^]^ Rye foods as compared to wheat have shown beneficial effects on insulin metabolism, increased satiety, decreased inflammation markers, and greater weight loss.^[^ ^9^ ^]^ Because of its high content of fiber and other components, such as a wide array of bioactive phytochemicals, rye may also positively affect gut health, possibly by modifying the composition and functionality of gut microbiota.^[^ ^10^, ^11^ ^]^ The changes in the microbial composition and function by diet have further implications for host health both via production of specific metabolites^[^ ^12^ ^]^ and via modulation of the immune responses,^[^ ^13^ ^]^ which opens new opportunities for improving health via personalized nutrition.

Rye is often consumed as sourdough fermented bread.^[^ ^9^ ^]^ Sourdough fermentation is a bread baking method that requires the presence of lactobacilli—either added as a starter or spontaneously appearing—to leaven the dough with yeasts. In addition to the food technological and organoleptic properties, sourdough may also have positive implications for human health by increasing the bioavailability of minerals, vitamins, and other bioactive components and by improving gut health via modulating the fiber matrix, producing probiotic exopolysaccharides, and potentially influencing the gut microbiota via LAB‐produced metabolites, even though the baked products no longer contain live bacteria.^[^ ^14^, ^15^, ^16^, ^17^ ^]^

Several compounds produced by lactic acid bacteria have been confirmed or suggested to have antimicrobial properties.^[^ ^18^, ^19^, ^20^, ^21^, ^22^ ^]^ These include small acidic metabolites and bacteriocins, which are peptides or small proteins with bacteriocidic effects. These compounds may modulate the microbial composition beneficially both in terms of the LAB strain survival and host health. However, single compounds used in isolation have not reached the antimicrobial activity observed when studying the inhibitory effect of the LAB strains themselves.^[^ ^23^, ^24^ ^]^ This suggests that a synergistic effect from several different bioactive molecules may be necessary to properly inhibit microbial growth. Limited research exists on the antimicrobial activity of LAB‐derived metabolites tested both separately and as a mixture to assess the potential synergistic effect. The information is scarce also on whether the same antimicrobial compounds produced by LAB strains present in fermented foods could also be produced by the gut microbiota during the digestion of these foods.

### 1.1 Aim and Objectives

The current research aimed to investigate whether it was possible to define a general metabolite pattern representing fermentation of high‐fiber rye, from ingredients to bread and their gut microbial fermentation. The aim was further to investigate if such metabolite pattern—individually or synergistically—could inhibit the growth of selected pathogenic bacteria, as a model of a potential beneficial health effect. We studied metabolites produced by *L. plantarum* DSMZ 13890, a strain growing particularly well on rye bran substrate, and used high‐fiber rye ingredients and bread with flour from different sources as well as during in vitro colonic fermentation of the corresponding foods. The specific objectives were 1) to characterize metabolites produced by *L. plantarum* DSMZ 13890 when grown in rye bran and 2) to determine whether these metabolites are produced during the baking of several different sourdough bread and also during in vitro colonic fermentation, and 3) to study in vitro the inhibitory effects of these metabolites, both individually and as a mixture, against the selected panel of bacterial species with different properties, including human commensal bacteria (including known and potential pathogens), clinical isolates, and environmental isolates.

## 2 Experimental Section

### 2.1 Fermentation of Rye with *L. plantarum*

*L. plantarum* DSMZ 13890 was cultured on De Man, Rogosa, and Sharpe agar (Oxoid, Thermo Scientific, Stockholm, Sweden) for 24 h at 37 °C, colonies were suspended in tap water to a concentration of ≈10^5^ cfu mL^−1^ and added to the four different combinations of rye bran and whey powder or lactose (**Table** **1**).

**Table 1:** Mixtures of rye bran, lactose, and whey used for in vitro fermentation (w/v) and LC‐MS analyses

Rye bran	Lactose	Whey powder
1%	0%	0%
1%	2%	0%
1%	0%	2%
1%	2%	2%

**Figure 1:**
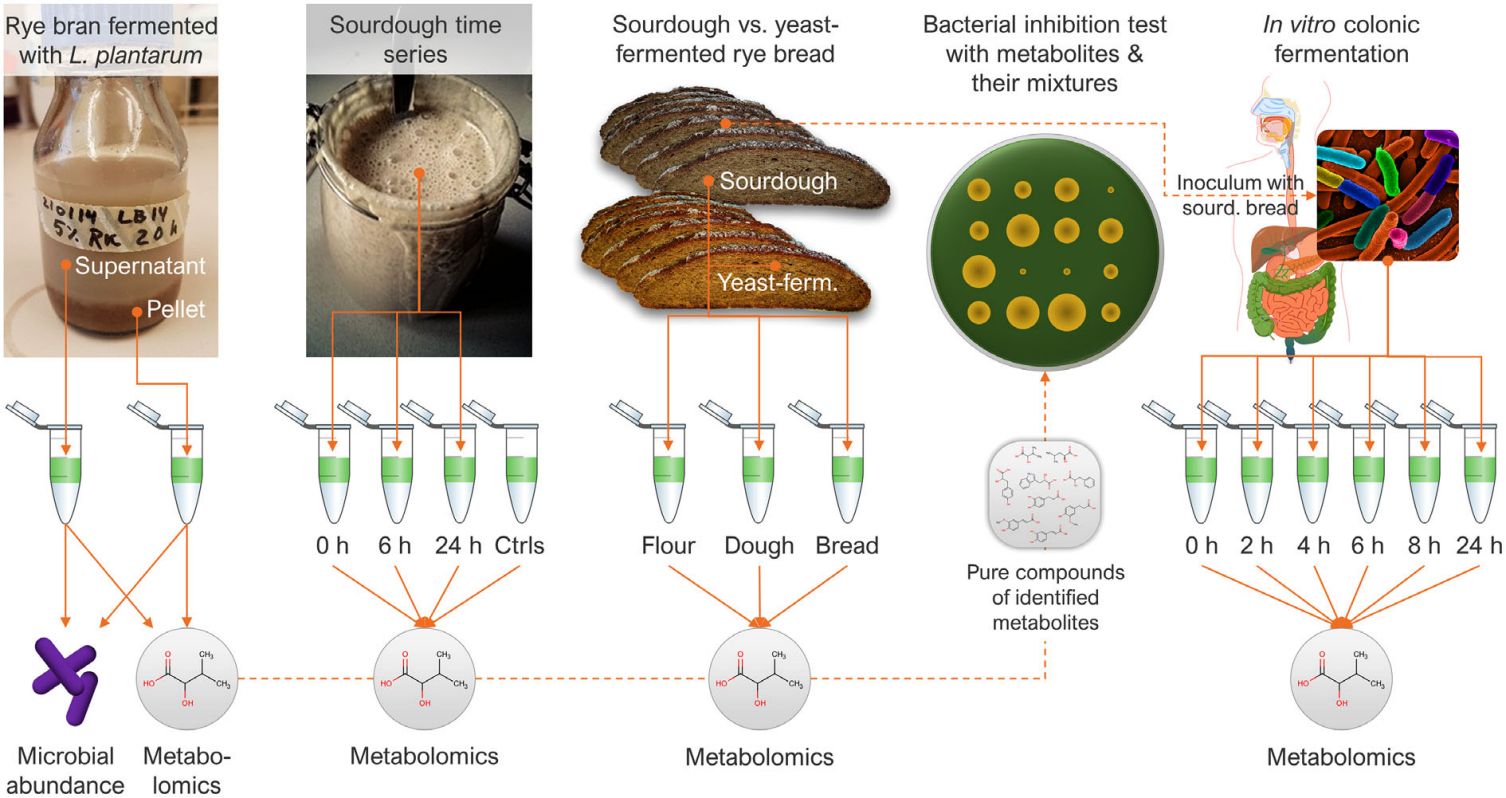
Schematic of the overall study design and individual experiments included in the study. The sample types and data acquired from the experiments are shown below each experiment.

The mixtures were incubated at 37 °C under gentle agitation and samples were collected after 1, 2, 4, 8, 16, 24, and 72 h. When incubated at 22 °C, collection of samples was done after 12, 24, and 72 h. The viable counts of *L. plantarum* (cfu mL^−1^) and pH were documented (Figure S2, Supporting Information) before the samples collected at different time points were centrifuged and stored at −80 °C, with the supernatant separated from the pellet. The overall study design was illustrated in **Figure** **1**.

### 2.2 Sourdough Fermentation

#### 2.2.1 Sourdough Bread

Regular sourdough whole‐grain rye bread and yeast‐fermented whole‐grain rye bread were prepared at VTT Technical Research Center of Finland as described earlier.^[^ ^15^ ^]^ Briefly, whole‐grain rye flour (Fazer Mill & Mixes, Finland) and a sourdough starter containing baker's yeast (*Candida milleri* C‐96250, 10^7^ cfu g^−1^), *Levilactobacillus brevis* (strain E‐95612, 10^8^ cfu g^−1^), and *L. plantarum* (strain E‐78076, 10^8^ cfu g^−1^) were used to produce the sourdough rye bread. The sourdough fermentation was performed at 32 °C for 20 h. The yeast‐fermented bread was made from the same flour without adding the lactobacilli into the starter.

#### 2.2.2 Sourdough Fermentation Time Series

Rye bran was fermented with *L. plantarum* DSMZ 13890 by mixing the bran with autoclaved tap water (1:5 w/w) and incubating at 37 °C for 24 h, as described in more detail by Xue et al.^[^ ^25^ ^]^ Samples were taken at the beginning of the fermentation, at 6 h, and at 24 h (end of the fermentation). The fermented rye bran, with a final concentration of *L. plantarum* DSMZ 13890 estimated at 10^9^ cfu mL^−1^, was then dried and incorporated into a tailor‐made rye crackerbread (WG rye with fermented rye bran).^[^ ^25^ ^]^ The reference products (unfermented and fermented rye crackerbread) were obtained from Wasabröd (Stockholm, Sweden).

### 2.3 Determination of Minimum Inhibitory Concentrations by Agar Dilution

MICs of the pure fermentation products, as well as the crude fermentate, were determined by the agar dilution technique.^[^ ^26^ ^]^ The bacteria were tested for their ability to grow on agar plates containing serial dilutions of the different substances of interest. The lowest concentration inhibiting visual growth was known as the MIC. Each substance was dissolved in a small amount of 100% methanol and further diluted in Milli‐Q water to reach the final concentration of 500 mM. A two‐fold serial dilution in Milli‐Q water was then performed (500, 250, 125, 62.5, and 31.25 mM) and 1 mL of each concentration was added to 19 mL melted Brucella agar (BBL), after which the plates were cast. The final concentrations in the assay ranged from 1.5 to 25 mM. MIC determinations of a mix containing the seven pure fermentation products at a concentration of 2.5 mM each, and another blend also including the two precursors ferulic and caffeic acid at 2.5 mM each, were also performed. The crude fermentate was treated similarly, resulting in concentrations from 0.3 to 5 vol% in the agar plates.

The panel of bacterial isolates selected for the inhibition test (Table S2, Supporting Information) was initially cultured on blood agar (Columbia agar base [Alpha, C03‐111A] + 5% defibrinated horse blood), grown colonies were suspended in phosphate‐buffered saline (PBS, 150 mM NaCl, pH 7.3), and transferred to the tray of a replicator used for inoculation of the test plates. One µL, about 10^5^ cfu per spot, of each strain was finally inoculated on the plates. Anaerobic bacteria were grown and tested at anaerobic conditions (Anoxomat, 5% H_2_, 10 % CO_2_ in N_2_) at 37 °C for 48 h, and the aerobic and facultative anaerobic bacteria in ambient air at 37 °C for 24–48 h. Growth control plates (blood agar and Brucella agar), without any antimicrobial substances added, were included in each test run.

Reference standards of 2‐hydroxyisocaproic acid (CAS 498‐36‐2), 3‐phenyllactic acid (CAS 828‐01‐3), 4‐hydroxyphenyllactic acid (CAS 306‐23‐0), 3‐indolelactic acid (CAS 832‐97‐3), α‐hydroxyisovaleric acid (CAS 17407‐56‐6), dihydroferulic acid (CAS 1135‐23‐5), dihydrocaffeic acid (CAS 1078‐61‐1), *trans*‐ferulic acid (CAS 537‐98‐4), and caffeic acid (CAS 331‐39‐5), were purchased from Sigma‐Aldrich.

### 2.4 In Vitro Colonic Fermentation

The whole‐grain test breads with sourdough or yeast fermentation were prepared for a previous study.^[^ ^15^ ^]^ The in vitro enzymatic digestion and fermentation of the bread by gut microbiota was performed following methods described by Aura et al.^[^ ^27^ ^]^ and Nordlund et al.^[^ ^28^ ^]^ Briefly, the breads were first treated with porcine enzymes (salivary α‐amylase, pepsin, and pancreatin), after which the digestion products were removed by dialysis. The remaining residues were freeze‐fried before adding them to the fecal material. The pooled fecal suspension (10% w/v) from five healthy volunteers was used in strictly anaerobic conditions for colonic fermentation, including a fecal sample without any added bread as a control. Samples were taken at 0, 2, 4, 8, and 24 h time points.

### 2.5 LC‐MS Analysis

For solid samples excluding the rye pellet (sourdough bread and sourdough fermented rye), the metabolite extraction was performed by adding 400 µL of cold 80% v/v aqueous methanol per 100 mg of frozen sample. The samples were sonicated at room temperature for 15 min and centrifuged at 13 000 rpm for 5 min, after which the supernatant was collected and filtered with Acrodisc CR 13 mm filters with a 0.2 µm PTFE membrane. For samples in suspension (in vitro colonic fermentation), 200 µL of cold 80% v/v aqueous methanol was added per 100 µL of the sample. The samples were vortexed and centrifuged (10 000 rpm, 5 min), and the supernatant was collected and filtered (Acrodisc CR 13 mm syringe filters with 0.2 µm PTFE membrane). For supernatant samples from rye fermented with *L. plantarum* DSMZ 13890, 900 µL of cold 90% v/v aqueous methanol was added per 100 µL of the sample. The samples were shaken vigorously for 3 min followed by centrifugation for 10 min at 4 °C (13 000 × *g*). For the pellet samples from rye fermented with *L. plantarum* DSMZ 13890, approximately 10 mg of each sample was weighed and extracted with 1 mL of cold 90 % v/v aqueous methanol. The pellet samples were then treated as the other solid samples above.

The pellet and supernatant from rye fermented with *L. plantarum* DSMZ 13890 were analyzed at Chalmers University of Technology with LC‐MS consisting of Agilent 1290 Infinity UHPLC coupled with Agilent 6520 Q‐TOF mass spectrometer. A Waters Acquity UPLC HSS T3 column (2.1 × 100 mm, 1.8 µm) kept at 45 °C was used for the reversed‐phase chromatographic separation. The eluents were HPLC grade water with 0.04% formic acid (eluent A) and HPLC grade methanol with 0.04% formic acid (eluent B). The gradient was as follows [*t* (min), %B]: [0, 5], [6, 100], [10.5, 100], [10.51, 5], [13, 5]. The ESI source was operated using the following conditions: gas (nitrogen) temperature of 175 °C and drying gas flow of 10 L min^−1^, nebulizer pressure of 45 PSI, a capillary voltage of 3500 V, fragmentor voltage of 125 V, a skimmer of 65 V, and octupole RFPeak at 750. For data acquisition, a 2‐GHz extended dynamic range mode was used, and the instrument was set to acquire data over the mass range of *m/z* 50–1700. Data were collected in centroid mode at an acquisition rate of 1.67 spectra s^−1^ with an abundance threshold of 200 counts. Continuous mass axis calibration was performed by monitoring two reference ions, *m/z* 121.050873 and 922.009798 for positive mode and *m*/*z* 112.988900 and 966.000725 for negative mode, from an infusion solution throughout the runs.

The sourdough fermented bread samples and the in vitro colonic fermented samples were analyzed at UEF with LC‐MS consisting of Agilent 1290 Infinity UHPLC coupled with Agilent 6540 Q‐TOF mass spectrometer with a method described in detail previously.^[^ ^29^ ^]^ An Agilent Zorbax Eclipse XDB‐C18 column (2.1 × 100 mm, 1.8 µm) was used for the reversed‐phase chromatographic separation. The eluents were HPLC grade water with 0.1% formic acid (eluent A) and HPLC grade methanol with 0.1% formic acid (eluent B). The gradient was as follows [*t* (min), %B]: [0, 2], [10, 100], [14.5, 100], [14.51, 2], [16.5, 2]. The sourdough time series with the reference samples were analyzed by Afekta Technologies, Ltd., using the same equipment and conditions.

### 2.6 Data Analysis and Statistics

The peak picking and metabolite annotation was performed separately for each LC‐MS dataset (fermented rye bread, fermented rye samples, rye pellet, and supernatant from the *L. plantarum* fermentation, and in vitro colonic fermented rye bread) with MS‐DIAL version 4.12 and later^[^ ^30^ ^]^ according to Klåvus et al.^[^ ^29^ ^]^ For data collection, MS1 tolerance was set at 0.01 Da, MS2 tolerance 0.025 Da, minimum peak height 2000 counts, mass slice width 0.1 Da, smoothing level three scans, and minimum peak width five scans. A database file containing the MoNA, RIKEN, MassBank, and UEF in‐house spectral databases was used as the reference. For peak alignment, retention time tolerance was set at 0.1 min, MS1 tolerance 0.015 Da, and “gap filling by compulsion” option was selected.

The fold change analysis (ratio of group averages) was performed in Microsoft Excel and the *p‐*values were corrected with Benjamini–Hochberg false discovery rate using an online calculator. The Pearson correlations with Benjamini–Hochberg FDR were calculated with the *notame* script^[^ ^29^ ^]^ using RStudio 1.1.447. Because the LC‐MS data were acquired from two independent platforms with slightly different chromatographic gradients, a regression model was utilized in Excel to merge the retention times and cross‐validate some of the metabolite annotations (see Figure S3, Table S3, Supporting Information).

## 3 Results and Discussion

### 3.1 Microbial Metabolites Produced by *L. Plantarum* Increased in Fermented Rye Bran and Bread

*L. plantarum* DSMZ 13890 was selected for the present in vitro tests due to excellent growth in rye bran (Warbro kvarn AB, Sköldinge, Sweden) suspensions as compared to other tested cereal products.^[^ ^31^ ^]^ After 24‐h incubation at 37 °C in a suspension of 5% rye bran in tap water, a pure culture of *L. plantarum* was grown, and bacterial cells were observed to adhere to different structures of rye bran (Figure S1, Supporting Information). In general, strains of *L. plantarum* showed better growth in mixtures of rye bran than other tested species in the *Lactobacillus* group: *Lacticaseibacillus casei, Lacticaseibacillus paracasei, Lacticaseibacillus rhamnosus, Lactiplantibacillus paraplantarum, Lactiplantibacillus pentosus, Lactobacillus helveticus*, and *Limosilactobacillus reuteri*.^[^ ^32^ ^]^ The growth conditions of *L. plantarum* DSMZ 13890 were optimized in different mixtures of rye bran, lactose, and whey (Table 1). A mixture of 1% rye bran, 2% lactose, and 2% whey showed to be suitable for promoting the growth of the strain determined by viable counts (colony forming units [cfu mL^−1^]), although rye bran alone, without supplements, was nearly as efficient (Figure S2, Supporting Information). The fermentation was performed at 37 °C and room temperature, showing a similar pattern at the two conditions, but with a slower pH drop and growth of *L. plantarum* at room temperature. The different substrate mixtures did not influence most of the *L. plantarum*‐related metabolites.

**Figure 2:**
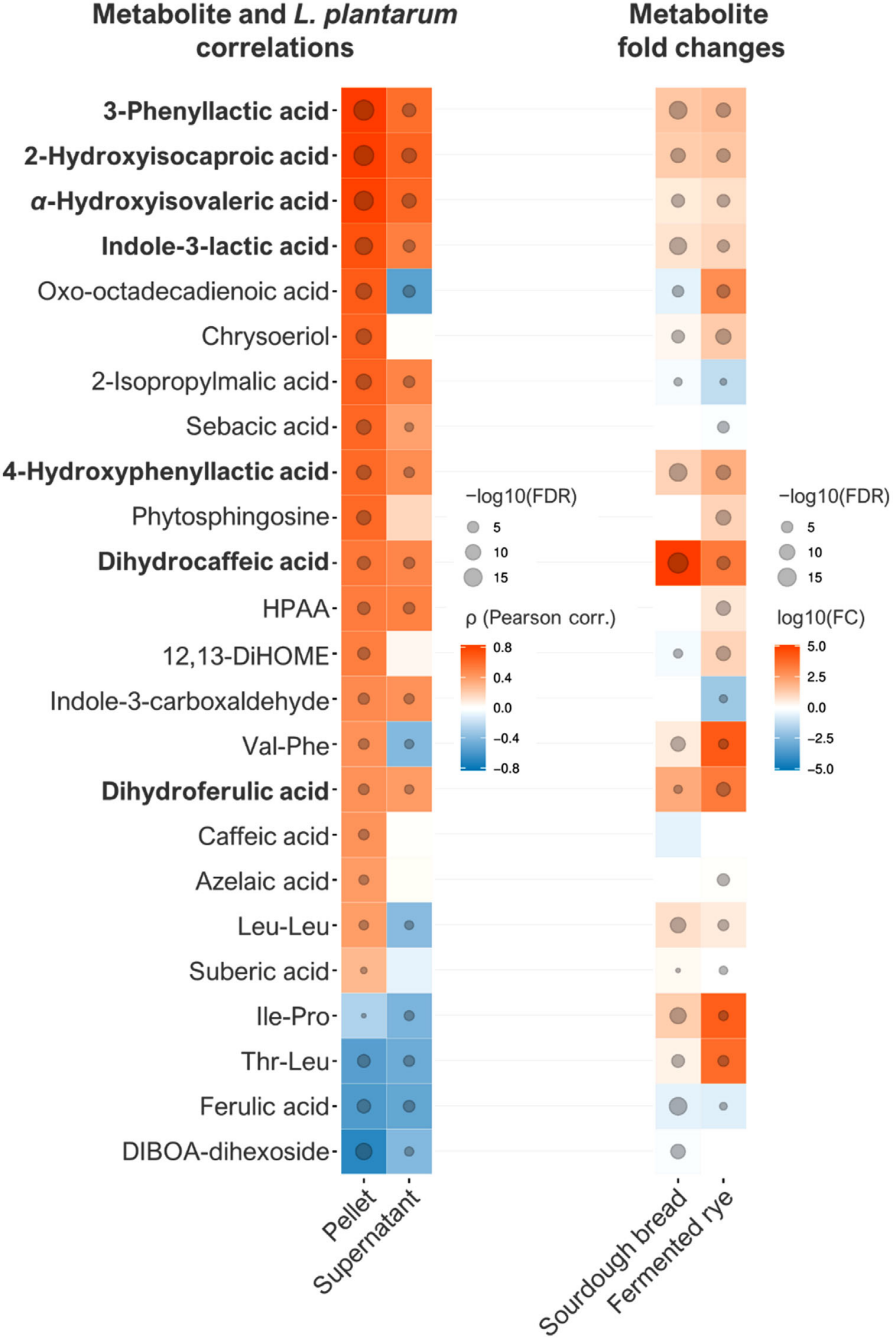
Heat map of the annotated metabolites. The left part of the heat map includes the correlations between the metabolites and *L. plantarum* abundance in the pellet and supernatant of rye bran fermented with the same bacteria. The right part shows the logarithmic fold changes of the metabolites in two rye fermentation studies: sourdough whole‐grain rye bread compared to its yeast fermented counterpart and a rye sample fermented for 24 h compared to an unfermented commercial whole‐grain rye crispbread. A log_10_(FC) value of 5 thus means a 100 000‐fold higher metabolite abundance compared to the control. The spheres represent the negative logarithm of the FDR‐corrected *p*‐value, and they are omitted for FDR values above 0.1. Metabolites typed in bold had a positive correlation (*ρ* > 0.4, FDR < 0.1) and a positive fold change (FC > 2, FDR < 0.1) in all the comparisons. HPAA, *N*‐(2‐Hydroxyphenyl)‐acetamide.

After analyzing the metabolites from the fermented rye pellet and supernatant samples with LC‐MS and determining the bacterial counts of the *L. plantarum* strain in the same samples, we identified or putatively annotated 24 metabolites correlating significantly (FDR < 0.1) with the levels of *L. plantarum* in either the fermented rye pellet or supernatant (**Figure** **2** and Table S1, Supporting Information). Out of these, 11 metabolites positively correlated with *L. plantarum* levels in both pellet and supernatant, including six microbial metabolites of amino acids, two microbial metabolites of phenolic acids, two dicarboxylic acids, and one phenylacetamide (HPAA). In addition to potentially selectively favoring the growth of lactobacilli, these water‐soluble compounds may represent bacterial metabolites that are available for absorption from the small intestine. If the production of these compounds from fermented foods continues in situ in the gut, they may favor the bacterial growth conditions of beneficial microbiota over pathogens in the gut.

The levels of four metabolites, including two dipeptides (Ile‐Pro and Thr‐Leu), one phenolic acid (ferulic acid), and one benzoxazinoid (DIBOA‐dihexoside) were negatively correlated with the *L. plantarum* levels in both pellet and supernatant. The dipeptides contain leucine and isoleucine, which are branched‐chain amino acid (BCAA) precursors of some of the positively correlated amino acid metabolites: leucine is the precursor of 2‐hydroxyisocaproic acid and all BCAAs are precursors of *α*‐hydroxyisovaleric acid. Similarly, ferulic acid is the precursor of dihydroferulic acid and DIBOA‐dihexoside that of HPAA [*N*‐(2‐Hydroxyphenyl)‐acetamide], suggesting that these compounds have been transformed by *L. plantarum* into microbial metabolites.

**Figure 3:**
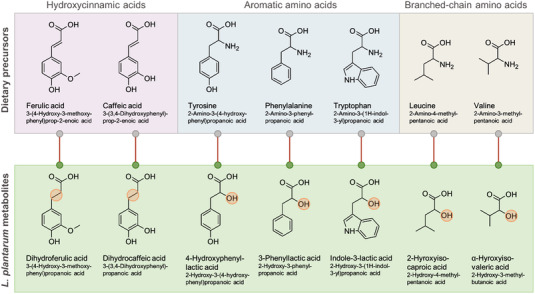
Structures of the seven metabolites commonly produced in the fermentation experiments and their precursors. Hydroxycinnamic acids experience reduction of the double bond whereas amino acids undergo deamination during the microbial metabolism (highlighted in the metabolite structures).

**Figure 4:**
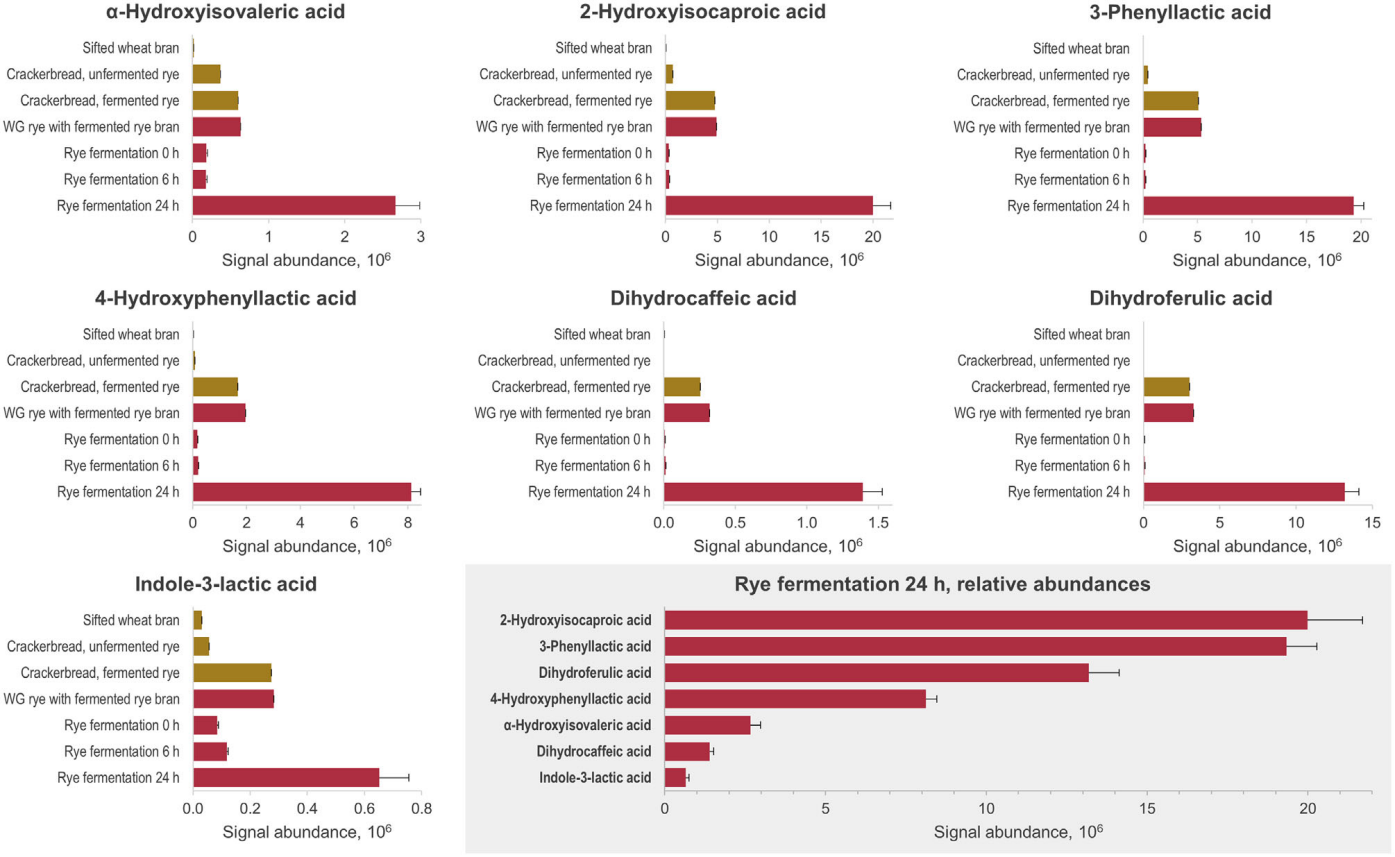
The levels (as peak areas) of *L. plantarum*‐associated metabolites in various reference samples (yellow bars) made of wheat (sifted wheat bran) or rye (regular whole‐grain rye crackerbread and fermented whole‐grain rye crackerbread) and in three parallel fermentations of rye bran with *L. plantarum* DSMZ 13890 at 0, 6, and 24‐h time points as well as a tailor‐made whole‐grain rye crackerbread fortified with the fermented rye bran (red bars).

To confirm the presence of the correlating metabolites in food products made of rye, we analyzed samples from whole‐grain rye bread fermented with traditional sourdough [containing baker's yeast (*C. milleri* C‐96250), *L. brevis* (strain E‐95612), and *Lactiplantibacillus plantarum* (strain E‐78076)] or plain baker's yeast and from a time series of rye bran fermentation, where *L. plantarum* DSMZ 13890 was used. Among the 11 metabolites positively correlated with *L. plantarum* in both pellet and supernatant of fermented rye, seven compounds also had significantly higher levels in fermented rye in both fermentation experiments, where sourdough whole‐grain rye bread was compared to its yeast‐fermented counterpart (Figure 2 and Table S1, Supporting Information). These compounds were metabolites of branched‐chain amino acids (2‐hydroxyisocaproic acid and *α*‐hydroxyisovaleric acid), aromatic amino acids (3‐phenyllactic acid, 4‐hydroxyphenyllactic acid, and 3‐indolelactic acid), and phenolic acids (dihydrocaffeic acid and dihydroferulic acid) (**Figure** **3**). In the fermentation of rye bran with lactobacilli, the levels of these metabolites remained low until the 6‐h time point, but they increased considerably at the end of the fermentation (**Figure** **4**). These findings verify that the compounds are present at high levels in different fermented foods prepared from whole‐grain rye, as compared to corresponding food products prepared without sourdough, or another type of fermentation involving LAB strains.

2‐Hydroxyisocaproic acid (2‐HICA, leucic acid) is the end product of leucine metabolism and a known metabolite of lactobacilli with antimicrobial and antifungal activity^[^ ^20^, ^33^, ^34^ ^]^; it contributes to the extended shelf life of fermented products^[^ ^35^ ^]^ and has previously shown potential in inhibiting bacterial growth within dental root canals^[^ ^36^ ^]^ and topically against a broad range of bacteria, including antibiotic‐resistant strains of *Staphylococcus aureus* and *Escherichia coli*.^[^ ^18^ ^]^ 2‐Hydroxyisocaproic acid and α‐hydroxyisovaleric acid (a metabolite of branched‐chain amino acids^[^ ^34^ ^]^), were found to inhibit the growth of both bacteria (*E. coli*) and fungi (Trichophyton) at the concentration of 1 mg mL^−1^ (complete inhibition at 4 mg mL^−1^), and although these concentrations are likely much higher than what could be expected for this individual metabolite in the gut, the effect was independent of the pH‐lowering effect of these acids.^[^ ^20^ ^]^ 3‐Phenyllactic acid, produced primarily by *L. plantarum* as well as some other lactobacilli and bifidobacteria,^[^ ^37^, ^38^ ^]^ has shown antimicrobial activity against Gram‐positive bacteria, such as *Enterococcus faecalis* and *S. aureus*, and Gram‐negative bacteria, including *Klebsiella oxytoca*, *Providencia stuartii*, and *Salmonella enterica*.^[^ ^21^, ^39^ ^]^ It is also an antifungal agent.^[^ ^38^ ^]^ 4‐Hydroxyphenyllactic acid is among the known antimicrobial agents produced by strains of Lactobacillus and Leuconostoc.^[^ ^22^ ^]^ 3‐Indolelactic acid is mainly produced by lactobacilli^[^ ^40^ ^]^ and bifidobacteria.^[^ ^41^ ^]^ 3‐Indolelactic acid and other indole‐derived microbial metabolites of tryptophan activate the aryl hydrocarbon receptor (AhR)‐dependant IL‐22 production, which improves mucosal homeostasis in the GI tract and protects it from damage.^[^ ^42^ ^]^ The antimicrobial activity of dihydroferulic acid has not been widely studied but it has shown activity against *Saccharomyces cerevisiae* (MIC_50_ 6.4 mM).^[^ ^43^ ^]^ Dihydrocaffeic acid showed a MIC of approximately 3.8 mM against *E. coli*, which was higher than that of its precursor caffeic acid (MIC ≈1.4 mM).^[^ ^44^ ^]^ Certain phenolic acids and their derivatives, such as phenethyl ester of caffeic acid^[^ ^45^ ^]^ and ferulic acid,^[^ ^46^ ^]^ may protect against *Helicobacter pylori*‐induced gastritis by inhibiting the activation of nuclear factor‐κB (NF‐κB), which would otherwise promote chronic inflammation in gastric cells. In addition, ferulic acid was shown to inhibit the growth of *Pseudomonas aeruginosa, E. coli*, *S. aureus*, and *Listeria monocytogenes* in concentrations ranging from 0.1 to 1.25 mg mL^−1^.^[^ ^47^ ^]^

### 3.2 Combination of Metabolites Boosts Their Antimicrobial Activity

The seven investigated metabolites were selected based on their correlation with the growth of *L. plantarum* and their potential antimicrobial activity reported earlier in the literature.^[^ ^18^, ^19^, ^20^, ^21^, ^36^, ^38^, ^39^ ^]^ We decided to investigate the antimicrobial activity of each metabolite, their combination with and without phenolic acid precursors, and the crude supernatant of rye bran fermented with *L. plantarum* DSMZ 13890, using agar dilution as the test method. The bacteria selected for examination were of different types: Gram‐positive, Gram‐negative, aerobic, facultatively anaerobic, obligately anaerobic, and originating from various locations: human oral and gastrointestinal isolates, clinical isolates, and bacteria collected from the environment (Table S2, Supporting Information). Out of the 27 investigated bacterial strains, 12 were exclusively environmental isolates.

**Figure 5:**
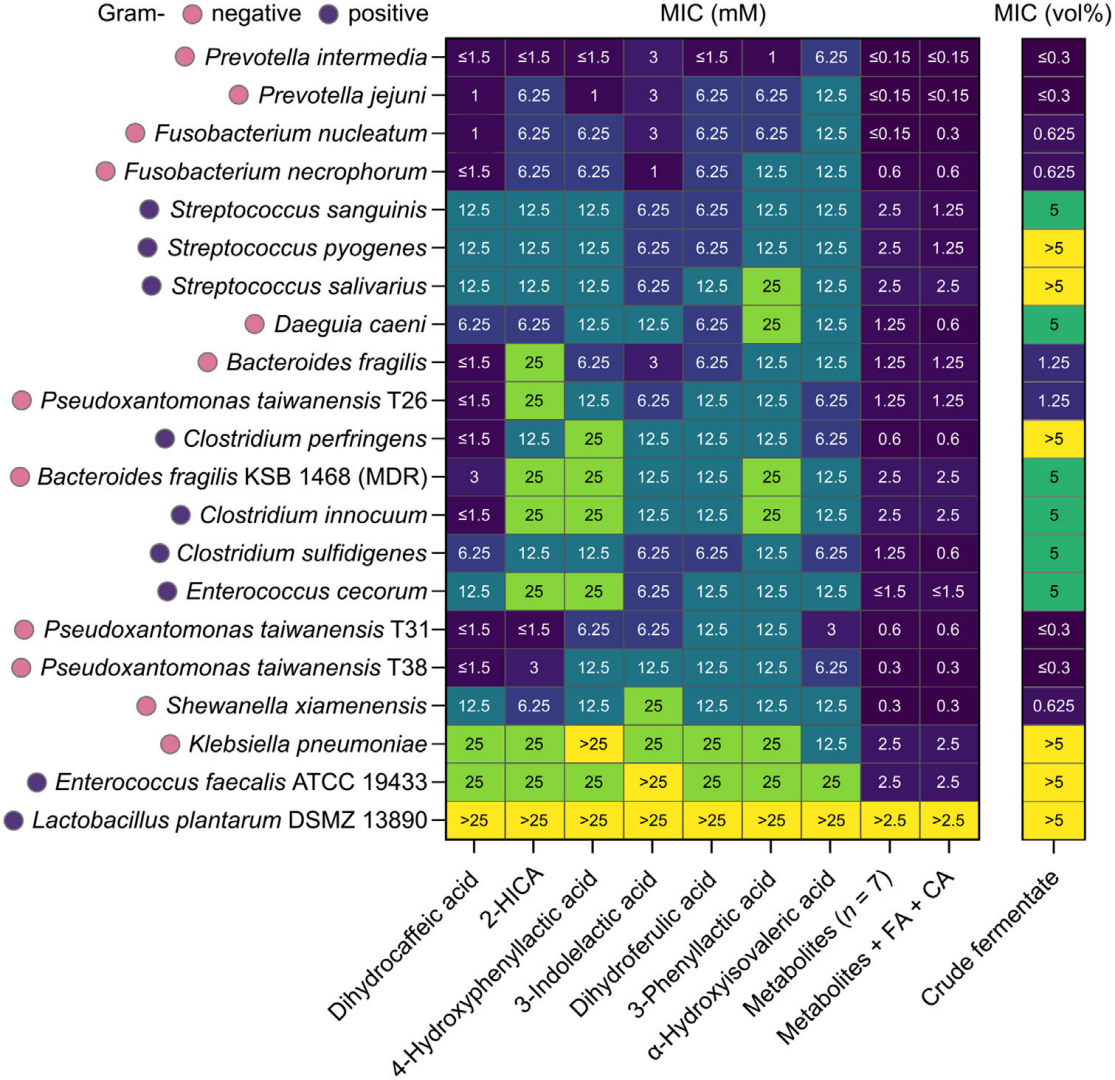
Unsupervised hierarchical clustering of the results from the agar dilution inhibition test. The minimum inhibitory concentration (MIC) was determined for seven potential *L. plantarum* metabolites, a mixture of the metabolites, and the metabolite mixture including two phenolic acid precursors abundant in whole grains (ferulic and caffeic acid; FA + CA) against a panel of Gram‐negative and Gram‐positive bacteria. The concentrations in the test ranged from 25 to 0.15 mM, and in the mixtures, each compound was diluted to the reported concentration. In addition, minimum inhibition is shown for the crude supernatant from the fermented rye bran containing the studied *L. plantarum* strain. The dilutions in the test ranged from 5% v/v (corresponding with crude fermentate:agar ratio 1:20) to 0.3% v/v (crude fermentate:agar ratio 1:320). Yellow color signifies no inhibitory effect at the highest concentration tested. Results for the seven environmental *Bacillus* strains are presented in Table S2, Supporting Information.

The results were clustered into a heatmap (**Figure** **5**) and assembled in Table S2, Supporting Information. The individual metabolites show a moderate to weak inhibition of the bacterial strains, MIC ranging from ≤1.5 (lowest tested concentration) to 25 mM (highest tested concentration). *L. plantarum* DSMZ 13890 was used as a negative control, and the tested concentrations showed no inhibition of the growth of the strain. Weak inhibition was also observed for *Klebsiella pneumoniae* and *E. faecalis*. In contrast, bacteria including opportunistic pathogen strains of *Prevotella* and *Fusobacterium* were inhibited at concentrations ranging from ≤1.5 to 12.5 mM. Differences in the MIC values were also observed between strains of the same bacterial species, such as for the response of the three strains of *Pseudoxantomonas taiwanensis* to 2‐HICA and the higher MIC of the multidrug‐resistant *Bacteroides fragilis* strain as compared to the antimicrobial‐susceptible type strain of the species. Dihydrocaffeic acid had a considerably stronger inhibition towards certain bacteria, such as both strains of *B. fragilis*, compared to the other tested metabolites. However, when the metabolites were combined into the agar as a mixture, the MICs drastically decreased for all the tested strains, with the lowest measured inhibitory concentrations at ≤0.15 mM for the two *Prevotella* strains. Again, the negative control strain *L. plantarum* was not inhibited by the highest tested concentration (2.5 mM). The addition of two phenolic acid precursors abundant in rye bran, ferulic and caffeic acid, may have moderately increased the inhibitory effect of the metabolite mixture against certain bacteria, but the data are not sufficient to verify this effect. Finally, we tested the crude supernatant of rye bran fermented with *L. plantarum* DSMZ 13890, which contains all the studied metabolites and any additional components released from the food matrix or produced in the lactobacilli fermentate, to inhibit bacterial growth. A somewhat similar inhibition pattern occurred, as compared to the tests with pure metabolites, displaying the strongest inhibition for *Prevotella*, *Fusobacterium*, and *Pseudoxantomonas*. Six strains, including the negative control, were not inhibited by the highest tested concentration (5% v/v). The results indicate that the relatively weak inhibitory effect of the individual *L. plantarum* metabolites significantly increases by combining the metabolites as a mixture, suggesting at least an additive, and in some bacteria, a synergistic effect. This can be estimated by comparing the average MIC values of the individual metabolites with the cumulative molarity of the metabolite mixtures. Importantly, many Gram‐negative bacteria are among the species that were most susceptible for the growth inhibition; they are more likely to develop antibiotic resistance compared to Gram‐positive bacteria.^[^ ^48^ ^]^

### 3.3 In Vitro Colonic Fermentation Reveals Production of the Antimicrobial Compounds

**Figure 6:**
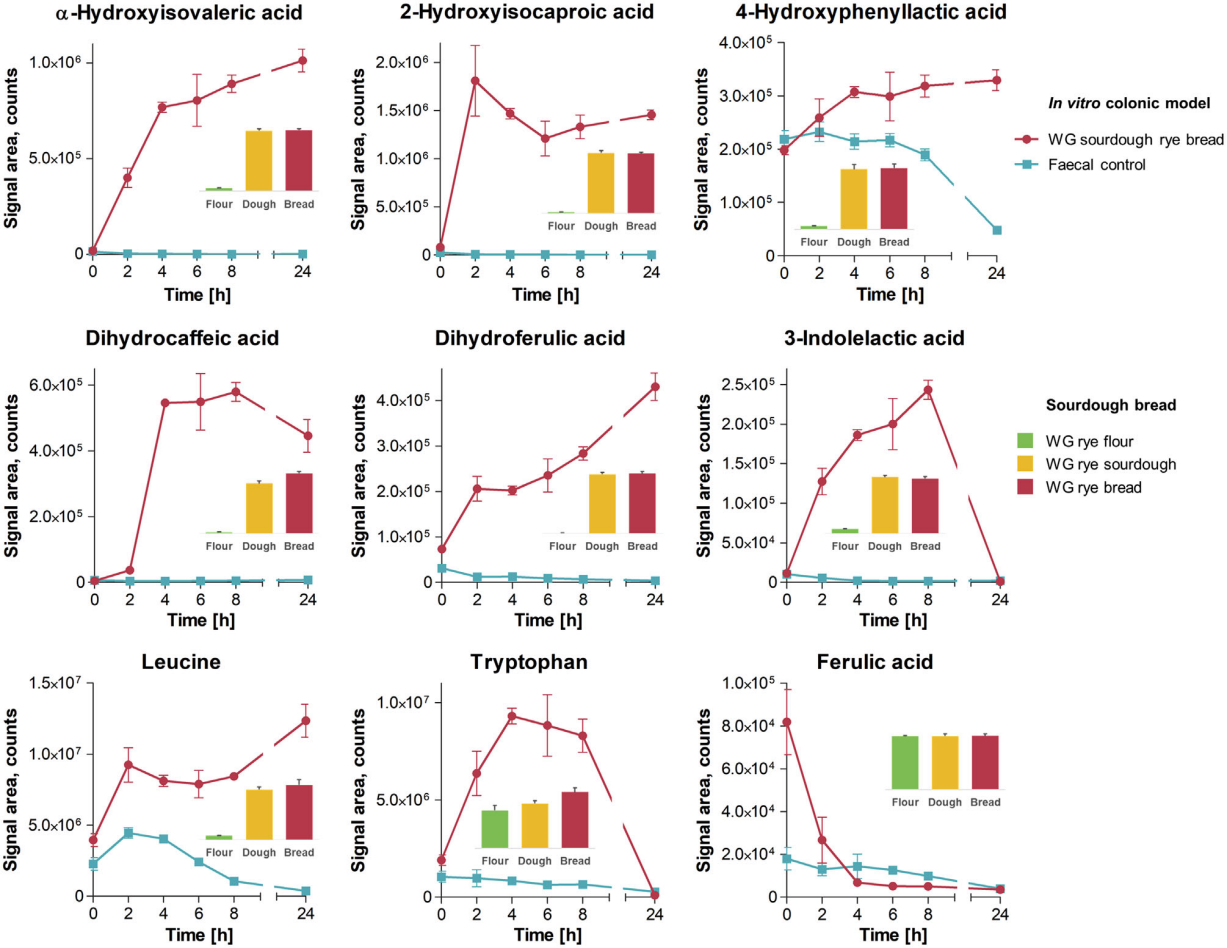
The levels of selected differential metabolites (α‐hydroxyisovaleric acid, 2‐hydroxyisocaproic acid, 4‐hydroxyphenyllactic acid, dihydrocaffeic acid, dihydroferulic acid, and 3‐indolelactic acid) and precursors (leucine, ferulic acid, and tryptophan) during the 24‐h in vitro colonic fermentation. The model was incubated with pooled human fecal material (used as fecal control) added with sourdough‐fermented whole‐grain rye bread (WG sourdough rye), both of which first underwent the upper intestinal model. In addition, the relative abundances of each metabolite are shown in whole‐grain rye flour and sourdough as well as bread prepared from the same flour (the same WG sourdough rye bread as in the colonic fermentation).

To study whether the seven potentially antimicrobial metabolites of *L. plantarum*, which were formed during the rye bran and rye bread fermentations, are also being produced in the human gut from ingested rye, we followed the course of the metabolites in the in vitro colonic fermentation of the same sourdough rye bread that we have analyzed previously^[^ ^15^ ^]^ (**Figure** **6**). The levels of *α*‐hydroxyisovaleric acid and dihydroferulic acid increased throughout the whole fermentation in both incubations, where sourdough fermented whole‐grain rye bread was added to the inoculum, while in the fecal background without added rye bread, the metabolites remained at trace levels. The metabolite levels in the incubations containing yeast‐fermented whole‐grain rye bread did not differ significantly from its sourdough fermented counterpart (data not shown). 2‐Hydroxyisocaproic acid, dihydrocaffeic acid, and 3‐indolelactic acid also increased significantly in the samples inoculated with rye during the 2–8 first hours of the fermentation, after which their levels remained the same or decreased while the fecal background remained in low or trace levels. The levels of 4‐hydroxyphenyllactic acid were similar for all three sample types at the beginning of the fermentation, with a moderate increase in the two rye inocula during the fermentation and a significant decrease in the fecal background. Of the precursors of the metabolites, we observed an increase in leucine and tryptophan levels both during the sourdough and in vitro colonic fermentation, while the levels of (free) ferulic acid were unaltered by sourdough and decreased rapidly to background level during the in vitro colonic fermentation (Figure 6). We were unable to determine the levels of 3‐phenyllactic acid in the samples due to its peak overlapping with another compound with an identical formula [3‐(3‐hydroxyphenyl)propionic acid] in the chromatography (data not shown).

The results of the in vitro colonic fermentation show that out of the seven metabolites associated with the antimicrobial activity of *L. plantarum*, at least six were also further produced when the sourdough fermented rye was incubated with colonic microbiota. *L. plantarum*, along with several other related LAB species, is a naturally present commensal bacterium in the human gut,^[^ ^49^ ^]^ and thus the production of these metabolites could be expected also in the gut. The increase in the amino acid precursor levels during both sourdough and in vitro colonic fermentation is best explained by the microbial breakdown of proteins and peptides into amino acids, which provides a constant source for the production of the microbial metabolites. In contrast, ferulic acid liberated from the fiber matrix by the gut microbiota seems to be rapidly and efficiently transformed into dihydroferulic acid, as indicated by the depletion of ferulic acid during the in vitro fermentation and the similarly fast increase in diferulic acid levels. The matrix of rye bran is particularly resistant for digestion, and therefore it is not surprising that a significant portion of the precursors survives after the sourdough fermentation and digestion of the upper gastrointestinal tract to be released and metabolized by the vast enzymatic capability of the gut microbiota. We thus hypothesize that these metabolites can act as mediators for the modulation of gut microbiota by *L. plantarum* and other related bacterial strains capable of producing the same metabolites.

The determination of the potential antimicrobial metabolites was based on correlating the relative abundances of metabolites from nontargeted metabolomics analyses with bacterial counts in the same samples. Therefore, the production of the metabolites by the studied strain remains to be verified with, for example, isotope‐labeled reference standards. In addition, as a general limitation inherent to nontargeted LC‐MS, it was not possible to perform quantitation or to identify all metabolites that correlated with the bacterial counts, and thus, the concentrations and the complete pool of potential antimicrobial metabolites remain to be determined. Lactic acid bacteria also produce bacteriocins, small antibacterial peptides shown to inhibit other bacteria, including potential pathogens.^[^ ^50^, ^51^ ^]^ Because bacteriocins have masses of several kilodaltons, they are outside the range of the LC‐MS system utilized in this study, and therefore their role in the bacterial inhibition in comparison to small metabolites was not studied.

It remains to be demonstrated whether the results obtained from in vitro experiments could be translated into in vivo conditions, given the relatively high concentrations of metabolites used in vitro. Axel et al.^[^ ^35^ ^]^ quantified antifungal carboxylic acids from wheat sourdough and reported concentrations up to 360 mg kg^−1^ (2.72 mmol kg^−1^) for 2‐hydroxyisocaproic acid and 194 mg kg^−1^ (1.17 mmol kg^−1^) for 3‐phenyllactic acid. However, dihydrocaffeic, dihydroferulic, and 4‐hydroxyphenyllactic acid had several folds lower concentrations or were not detected at all in some of the sourdough fermentations,^[^ ^52^ ^]^ which means that their potential to exert observable impact on oral microbiota, for instance, is limited. Because rye sourdough is commonly fermented for a longer time and at a higher temperature as compared to wheat, the metabolite concentrations may therefore be higher in fermented rye compared to wheat.^[^ ^15^ ^]^ In addition, the concentrations of the compounds in sourdough do not necessarily reflect the concentrations reached in the gut because of the more extensive metabolism performed by gut microbiota. The concentrations of any of the seven metabolites discussed herein have previously not been reported from fecal samples after cereal intake or from in vitro colonic fermented cereal material, and data are very limited on the concentrations altogether in microbial incubations. In addition, the intestinal secretion and the absorption of the metabolites may affect the final concentrations, which make it more challenging to speculate the actual in vivo concentration at the potential site of action. Rechner et al.^[^ ^53^ ^]^ observed dihydrocaffeic acid concentrations ranging from 50 to 600 mg L^−1^ (0.27–3.3 mM) in fecal material from various donors after an in vitro incubation with a dietary precursor compound (chlorogenic acid). Beloborodova et al.^[^ ^54^ ^]^ incubated intestinal microbes in a broth mimicking intestinal conditions and reported phenyllactic acid concentrations of 0.27 and 0.48 mM as well as 4‐hydroxyphenyllactic acid concentrations of 0.08 and 0.21 mM produced by *Limosilactobacillus fermentum* and *Bifidobacterium bifidum*, respectively. These concentrations, while not reaching the MICs of the individual tested metabolites, are within the tested MICs of the metabolite mixtures against several bacterial strains in the current study (Figure 5). This indicates that the concentrations of these compounds reached in the gut may be sufficient to induce a similar inhibitory effect as seen in vitro, considering also that more metabolites than the seven compounds characterized in this study may contribute to the inhibitory effect. However, more quantitative data are required to make further conclusions. The strict distribution of *L. plantarum* cells to the different rye bran structures upon fermentation may also indicate the occurrence of a microenvironment with substantially enhanced metabolite concentrations (Figure S1, Supporting Information).

## 4 Concluding Remarks

Seven microbial metabolites originating from amino acid and phenolic acid precursors were associated with the studied *L. plantarum* strain, and their production was observed in typical LAB fermentations as well as during the in vitro colonic fermentation of whole‐grain rye bread. We found a significantly stronger inhibition of several potentially harmful bacterial species—including antibiotic‐resistant Gram‐negative bacteria isolated from humans and the environment—when applying the mixture of the seven metabolites, as compared to the minimum inhibitory concentrations of the individual metabolites. These results support the hypothesis that metabolites from dietary sources work in synergy to exert local or systemic modulatory effects and that microbiota present both in fermented foods and in the gut contribute to the modulation of the microbial ecology in the gastrointestinal tract. It furthermore suggests that it is possible to identify a general metabolite profile from *L. plantarum* DSMZ 13890 fermentation of different types of rye ingredients and products and their in vitro gut fermentation that may have beneficial implications for gut and host health. The potential of rye as a prebiotic for the growth of beneficial gut bacteria, such as lactobacilli and bifidobacteria, is highlighted by the results, suggesting further implications for the modulation of gut and host health. Further studies are warranted on the subject, including quantitation of the metabolites from bread and colonic content as well as trials with human subjects.

## Conflict of Interest

K.H. is affiliated with Afekta Technologies, Ltd, all other authors declare no conflict of interest.

## Author Contributions

K.H. and R.L. Shared last authorship. V.M.K. performed the metabolomics data analysis and prepared the manuscript. M.H. and A.J. carried out the MIC agar dilution tests. L.S. contributed to the metabolomics data analysis and fermentation experiments. O.S. and M.L. were responsible for the LC‐MS analysis of the samples. A.M.A. designed and oversaw the colonic fermentation experiment with the in vitro digestion model. K.H. and R.L. coordinated the project collaboration and the overall study design. All authors critically reviewed the manuscript.

## Supporting information

Figure S1. Microscopic image of Gram‐stained cells of Lactiplantibacillus plantarum DSMZ 13980 cultured for 24 h at 37 °C in 5 % w/v of rye bran in water (magnification 1000×).
Figure S2. Registrations of changing in L. plantarum growth (cfu/mL) (A & C) and pH changes (B & D) upon fermentations at 37 °C (A & B) or room temperature (C & D).
Figure S3. Linear regression model of the retention times of 29 metabolites detected in both analytical platforms.
Table S1. The Pearson correlations of the annotated metabolites with the L. plantarum abundance in the pellet and supernatant of the fermented rye samples and the fold changes of the same compounds in the sourdough bread samples and the rye fermentation experiment.
Table S2. Results from the in vitro agar dilution bacterial inhibition test (n/t = not tested).
Table S3. The observed m/z and retention times of the significantly correlating metabolites with L. plantarum (in pellet or supernatant) and the predicted retention times for the UEF platform, calculated from the observed RT in Chalmers data.
Table S4. Other characteristics of the significantly correlating metabolites with L. plantarum, including universal identifiers, level of identification, and main observed MS/MS fragments.

## Data Availability

The data utilized for this study is available in the supporting information.
