# Supplementary material for: Metabolite Pattern Derived from Lactiplantibacillus plantarum—Fermented Rye Foods and In Vitro Gut Fermentation Synergistically Inhibits Bacterial Growth
Source: Mol Nutr Food Res. 2022 Aug 19;66(21):2101096. doi: 10.1002/mnfr.202101096 (PMC9787878; doi:10.1002/mnfr.202101096)
Supplement: Supplementary file 1 — Figure S1. Microscopic image of Gram‐stained cells of Lactiplantibacillus plantarum DSMZ 13980 cultured for 24 h at 37 °C in 5 % w/v of rye bran in water (magnification 1000×). Figure S2. Registrations of changing in L. plantarum growth (cfu/mL) (A & C) and pH changes (B & D) upon fermentations at 37 °C (A & B) or room temperature (C & D). Figure S3. Linear regression model of the retention times of 29 metabolites detected in both analytical platforms. Table S1. The Pearson correlations of the annotated metabolites with the L. plantarum abundance in the pellet and supernatant of the fermented rye samples and the fold changes of the same compounds in the sourdough bread samples and the rye fermentation experiment. Table S2. Results from the in vitro agar dilution bacterial inhibition test (n/t = not tested). Table S3. The observed m/z and retention times of the significantly correlating metabolites with L. plantarum (in pellet or supernatant) and the predicted retention times for the UEF platform, calculated from the observed RT in Chalmers data. Table S4. Other characteristics of the significantly correlating metabolites with L. plantarum, including universal identifiers, level of identification, and main observed MS/MS fragments. [file MNFR-66-2101096-s001.pdf]

Supplementary material

Metabolite pattern derived from *Lactiplantibacillus plantarum*–fermented rye foods and *in vitro* gut fermentation synergistically inhibits bacterial growth

Ville M Koistinen[1,2,3]*, Maria Hedberg[4], Lin Shi[5,6], Anders Johansson[4], Otto Savolainen[1,5], Marko Lehtonen[7], Anna-Marja Aura[8], Kati Hanhineva[2,5,1≠], Rikard Landberg[5≠]

1. Institute of Public Health and Clinical Nutrition, University of Eastern Finland, Finland
2. Food Chemistry and Food Development Unit, Department of Biochemistry, University of Turku, Finland
3. Afekta Technologies Ltd, Kuopio, Finland
4. Department of Odontology/Oral Microbiology, Umeå University, Sweden
5. Division of Food and Nutrition Science, Department of Biology and Biological Engineering, Chalmers University of Technology, Gothenburg, Sweden
6. College of Food Engineering and Nutritional Science, Shaanxi Normal University, Xi'an, China
7. School of Pharmacy, University of Eastern Finland, Kuopio, Finland
8. VTT Technical Research Centre of Finland Ltd, Espoo, Finland

≠ Shared last authorship

* Corresponding author: Ville Koistinen, Institute of Public Health and Clinical Nutrition, University of Eastern Finland, Yliopistonranta 1, P.O. Box 1627, FI-70211 Kuopio, Finland; ville.m.koistinen@uef.fi

Merging LC-MS data from two analytical platforms

Because we acquired LC-MS data from two separate analytical platforms, it was necessary to perform the data analysis separately for the datasets. However, the methods used in the platforms differed only for the length of the gradient (13.0 min in Chalmers and 16.5 min in UEF) and the model of reversed-phase column used (both silica-based C18 columns with dimensions of 2.1 × 100 mm and 1.8 μm particle size) for the liquid chromatography. Therefore, to facilitate the interpretation of the two datasets and to increase the number of annotations, we performed a linear regression model on the retention times of 29 metabolites already annotated in both platforms. A strong correlation was found between the retention times ($R^2 = 0.9973$), extending from L-phenylalanine (RT about 1.95 min in both platforms) to heneicosenylresorcinol (RT 7.74 min in Chalmers and 11.43 min in UEF) (**Figure S3**). The maximum error of the model to the actual retention times of the 29 metabolites was 0.29 min in the UEF data, which can be considered acceptable for a tentative annotation of metabolites across the platforms. Using the retention time prediction from the regression model, we did a targeted search on the UEF data based on unknown molecular features with significant correlation in the Chalmers data. We annotated 24 metabolites that had a significant correlation between the abundance of *L. plantarum* in the pellet or supernatant of fermented rye. The compounds are listed in **Table S1**. Some of the metabolites in the Chalmers data were annotated based on MS/MS data available from a peak in the UEF data with the same *m/z* and the retention time within 0.3 minutes of the predicted value.

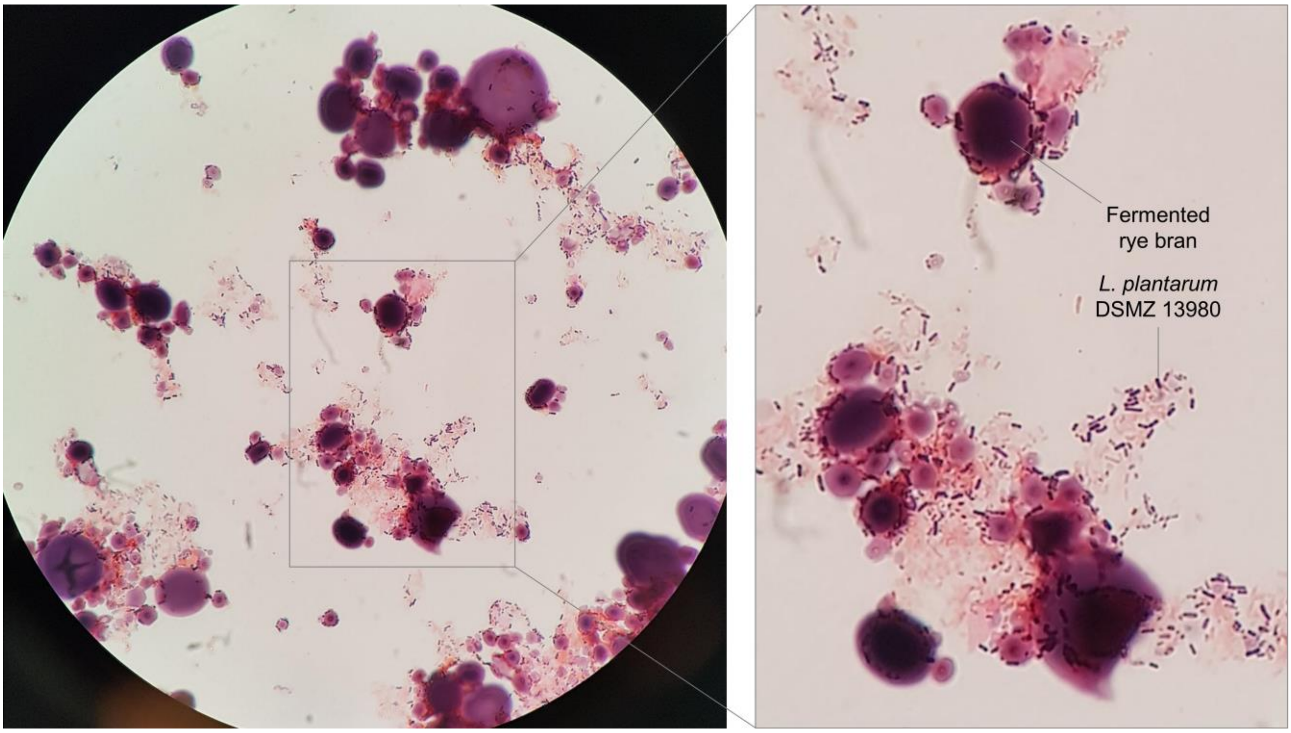

Figure S1. Microscopic image of Gram-stained cells of *Lactiplantibacillus plantarum* DSMZ 13980 cultured for 24 h at 37 °C in 5 % w/v of rye bran in water (magnification 1000×).

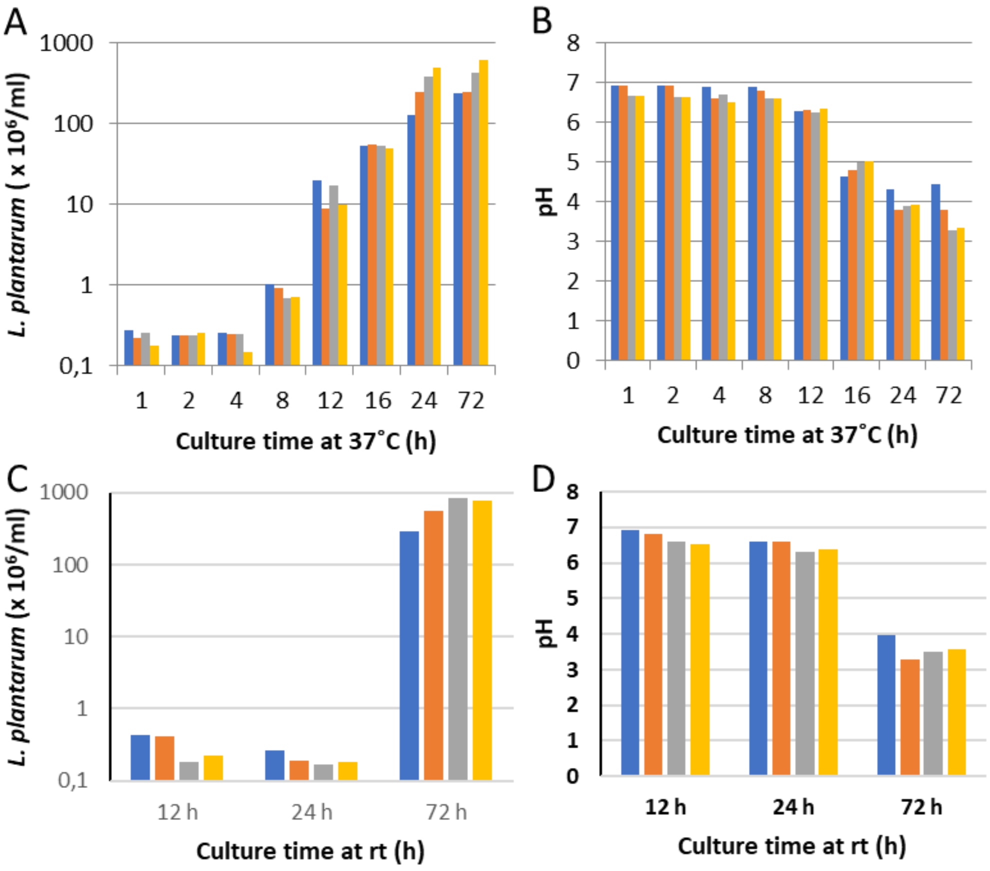

Figure S2. Registrations of changing in *L. plantarum* growth (cfu/mL) (A & C) and pH changes (B & D) upon fermentations at 37 °C (A & B) or room temperature (C & D). All mixtures contained *L. plantarum* 10^5/mL and 1 % (w/v) rye bran in tap water and different supplements. Bars in blue = rye bran and *L. plantarum*, orange = rye bran, *L. plantarum* and lactose (2 %), grey = rye bran, *L. plantarum* and whey (2 %), yellow = rye bran, *L. plantarum*, lactose (2 %) and whey (2 %).

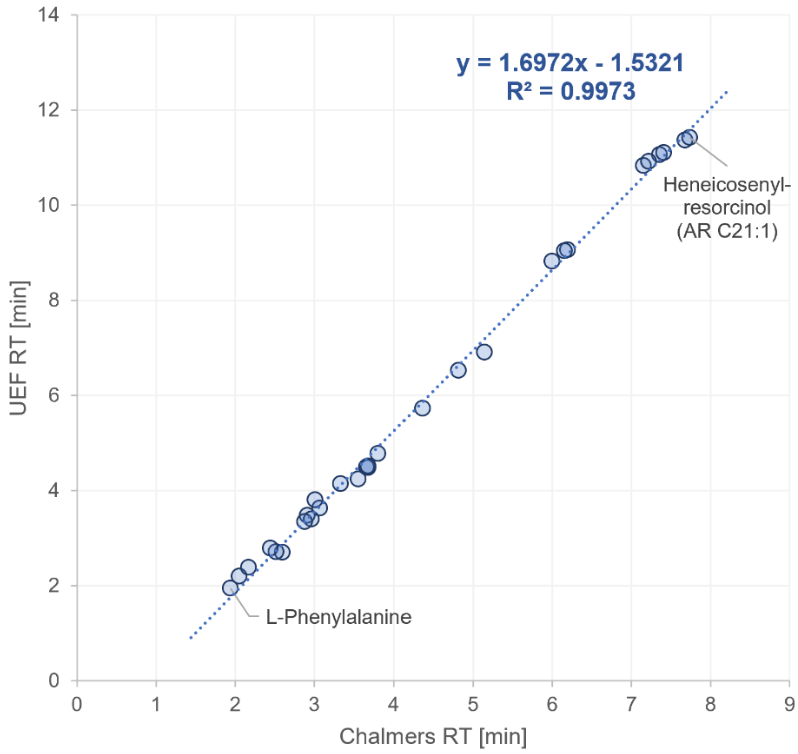

Figure S3. Linear regression model of the retention times of 29 metabolites detected in both analytical platforms.

Tables

Table S1. The Pearson correlations of the annotated metabolites with the *L. plantarum* abundance in the pellet and supernatant of the fermented rye samples and the fold changes of the same compounds in the sourdough bread samples and the rye fermentation experiment. The fold change is the ratio of the average signal abundance between the sourdough and yeast fermented rye bread (fold change > 1 means a higher level in sourdough bread). Statistically significant correlations and fold changes (FDR < 0.05) are typed in bold. The fold changes for some of the sourdough bread metabolites with raw *p*-values were published previously [15]. Metabolites marked with an asterisk were chosen for the *in vitro* inhibition test.

		Pearson correlation, pellet		Pearson correlation, supernatant		Sourdough vs yeast-fermented rye bread		Fermented rye vs unfermented rye crackerbread	
Curated ID	Description	ρ	FDR	ρ	FDR	Fold change	FDR	Fold change	FDR
3-Phenyllactic acid*	Phenylalanine metabolite	**0.83**	8.66E-20	**0.60**	4.25E-08	**27.6**	1.27E-14	**43.5**	3.6E-09
2-Hydroxyisocaproic acid*	Leucine metabolite	**0.82**	2.11E-19	**0.65**	7.41E-10	**20.2**	1.99E-09	**27.9**	5.3E-08
α-Hydroxyisovaleric acid*	BCAA metabolite	**0.80**	8.23E-18	**0.63**	3.25E-09	**4.0**	2.17E-07	**7.3**	6.9E-07
Indole-3-lactic acid*	Tryptophan metabolite	**0.73**	4.04E-14	**0.53**	2.21E-06	**5.8**	1.43E-13	**11.5**	2.4E-06
Oxo-octadecadienoic acid	Oxylipin	**0.68**	1.22E-11	**−0.54**	2.11E-06	**0.25**	1.33E-05	**769.7**	1.6E-07
Chrysoeriol	Flavonoid	**0.66**	1.07E-10	0.02	0.88	**2.1**	3.64E-07	**21.9**	2.4E-10
2-Isopropylmalic acid	Dicarboxylic acid	**0.66**	1.05E-10	**0.51**	1.03E-05	**0.53**	0.0041	**0.1**	0.015
Sebacic acid	Dicarboxylic acid	**0.63**	6.81E-10	**0.39**	0.0012	1.0	1	**0.6**	6.6E-06
4-Hydroxyphenyllactic acid*	Tyrosine metabolite	**0.62**	1.49E-09	**0.47**	7.37E-05	**13.9**	1.35E-14	**104.8**	1.9E-09
Phytosphingosine	Sphingolipid	**0.62**	2.89E-09	0.17	0.21	0.92	0.88	**14.0**	6.1E-10
Dihydrocaffeic acid*	Phenolic acid metabolite	**0.56**	1.00E-07	**0.50**	1.30E-05	**125320**	1.42E-19	**2345.3**	9.6E-08
N-(2-Hydroxyphenyl)-acetamide (HPAA)	Phenylacetamide	**0.54**	4.67E-07	**0.52**	6.00E-06	0.94	0.52	**4.7**	3.8E-09
12,13-DiHOME	Oxylipin	**0.53**	1.07E-06	0.05	0.72	**0.49**	0.0011	**13.3**	3.8E-09
Indole-3-carboxaldehyde	Indole	**0.48**	1.42E-05	**0.45**	1.52E-04	0.91	1	**0.0**	0.0046
Val-Phe	Dipeptide	**0.45**	5.28E-05	**−0.40**	8.27E-04	**3.9**	2.19E-09	**17780.1**	0.00027
Dihydroferulic acid*	Phenolic acid metabolite	**0.45**	4.44E-05	**0.41**	7.98E-04	**136.0**	0.0025	**2198.6**	1.6E-08
Caffeic acid	Phenolic acid	**0.44**	8.19E-05	0.02	0.90	0.27	0.100	0.9	0.73
Azelaic acid	Dicarboxylic acid	**0.41**	3.33E-04	0.03	0.83	1.1	0.53	**1.4**	2.1E-06
Leu-Leu	Dipeptide	**0.40**	4.92E-04	**−0.39**	0.0013	**6.5**	9.76E-11	**3.7**	3.8E-05
Suberic acid	Dicarboxylic acid	**0.29**	0.017	−0.09	0.55	**1.8**	0.033	**0.9**	0.0025
Ile-Pro	Dipeptide	**−0.26**	0.033	**−0.43**	3.11E-04	**18.4**	8.28E-12	**13152.7**	0.00038
Thr-Leu	Dipeptide	**−0.55**	3.05E-07	**−0.48**	3.40E-05	**2.5**	3.74E-07	**5986.0**	3.9E-05
Ferulic acid	Phenolic acid	**−0.58**	3.45E-08	**−0.51**	7.49E-06	**0.26**	2.49E-14	**0.2**	0.0067
DIBOA-dihexoside	Benzoxazinoid	**−0.70**	2.33E-12	**−0.40**	8.78E-04	**0.61**	1.74E-09	1.0	0.73

Table S2. Results from the *in vitro* agar dilution bacterial inhibition test (n/t = not tested). * Potential synergistic effect of the metabolite mixture based on the MIC difference of the average of the individual metabolite exposures and the cumulative molarity of the metabolites in the mixture (*i.e.*, MIC multiplied with the number of metabolites in the mixture, each diluted to the given concentration).

	MIC (mM)									MIC (vol%)
**Strain**	**2-HICA**	**Dihydro-caffeic acid**	**3-Indole-lactic acid**	**4-Hydroxy-phenyllactic acid**	**3-Phenyllactic acid**	**Dihydro-ferulic acid**	***α*-Hydroxy-isovaleric acid**	**Mixture (*n* = 7)**	**Mixture (*n* = 9)**	**Fermented rye bran supernatant**
Human isolates										
Streptococcus sanguinis	12.5	12.5	6.25	12.5	12.5	6.25	12.5	2.5	1.25	5
S. salivarius	12.5	12.5	6.25	12.5	25	12.5	12.5	2.5	2.5	>5
S. pyogenes	12.5	12.5	6.25	12.5	12.5	6.25	12.5	2.5	1.25	>5
Enterococcus faecalis ATCC 19433	25	25	>25	25	25	25	25	2.5	2.5	>5
Prevotella intermedia ATCC 25611	≤1.5	≤1.5	3	≤1.5	≤1.5	≤1.5	6.25	≤0.15*	≤0.15*	≤0.3
P. jejuni DSMZ 26989	6.25	≤1.5	3	≤1.5	6.25	6.25	12.5	≤0.15*	≤0.15*	≤0.3
Fusobacterium nucleatum	6.25	≤1.5	3	6.25	6.25	6.25	12.5	≤0.15*	0.3*	0.625
F. necrophorum	6.25	≤1.5	≤1.5	6.25	12.5	6.25	12.5	0.6*	0.6	0.625
Bacteroides fragilis ATCC 25285	25	≤1.5	3	6.25	12.5	6.25	12.5	1.25	1.25	1.25
B. fragilis KSB 1468	25	3	12.5	25	25	12.5	12.5	2.5	2.5	5
Clostridium innocuum	25	≤1.5	12.5	25	25	12.5	12.5	2.5	2.5	5
C. perfringens	12.5	≤1.5	12.5	25	12.5	12.5	6.25	0.6*	0.6*	>5
Lactiplantibacillus plantarum DSMZ 13890	>25	25	>25	>25	>25	>25	>25	>2.5	>2.5	>5
Primarily environmental										
Bacillus spp. (*n* = 7)	≤6.25–50	12.5	12.5–50	12.5	12.5–25	6.25–25	6.25–25	n/t	n/t	0.3–5
Clostridium sulfidigenes	12.5	6.25	6.25	12.5	12.5	6.25	6.25	1.25	0.6*	5
Daeguia caeni	6.25	6.25	12.5	12.5	25	6.25	12.5	1.25	0.6*	5
E. cecorum	25	12.5	6.25	25	12.5	12.5	12.5	1.25*	1.25	5
Klebsiella pneumoniae	25	25	25	>25	25	25	12.5	2.5	2.5	>5
Pseudoxantomonas taiwanensis T26	25	≤1.5	6.25	12.5	12.5	12.5	6.25	1.25	1.25	1.25
P. taiwanensis T31	≤1.5	≤1.5	6.25	6.25	12.5	12.5	3	0.6	0.6	≤0.3
P. taiwanensis T38	3	≤1.5	12.5	12.5	12.5	12.5	6.25	0.3*	0.3*	≤0.3

Table S3. The observed *m/z* and retention times of the significantly correlating metabolites with *L. plantarum* (in pellet or supernatant) and the predicted retention times for the UEF platform, calculated from the observed RT in Chalmers data. The metabolites marked with an asterisk were manually aligned using retention time prediction and putatively annotated in both datasets with MS/MS data from the UEF dataset.

		Observed values (Chalmers)		Observed values (UEF)		Retention time prediction (Chalmers to UEF)	
Curated ID	Ion	m/z	RT [min]	m/z	RT [min]	Predicted RT [min]	Error [min]
α-Hydroxyisovaleric acid	$[M-H]^-$	117.0555	2.57	117.0554	2.70	2.83	0.13
2-Hydroxyisocaproic acid	$[M-H]^-$	131.0714	3.57	131.0708	4.25	4.53	0.28
Indole-3-carboxaldehyde*	$[M-H]^-$	144.0455	3.81	144.0458	4.65	4.94	0.29
N-(2-Hydroxyphenyl)-acetamide (HPAA)*	$[M-H]^-$	150.0561	2.78	150.0556	3.28	3.19	−0.09
3-Phenyllactic acid	$[M-H]^-$	165.0557	3.69	165.0552	4.49	4.73	0.24
Suberic acid	$[M-H]^-$	173.0817	3.82	173.0816	4.78	4.95	0.17
2-Isopropylmalic acid	$[M-H]^-$	175.0610	2.98	175.0607	3.40	3.53	0.13
Caffeic acid*	$[M-H]^-$	179.0353	2.99	179.0341	3.41	3.55	0.14
Dihydrocaffeic acid*	$[M-H]^-$	181.0504	2.87	181.0504	3.22	3.34	0.12
4-Hydroxyphenyllactic acid	$[M-H]^-$	181.0506	2.55	181.0502	2.72	2.79	0.07
Azelaic acid	$[M-H]^-$	187.0972	4.37	187.0972	5.73	5.89	0.16
Ferulic acid	$[M-H]^-$	193.0510	3.70	193.0506	4.52	4.74	0.22
Dihydroferulic acid*	$[M-H]^-$	195.0658	3.56	195.0664	4.37	4.50	0.13
Sebacic acid	$[M-H]^-$	201.1124	4.83	201.1127	6.53	6.67	0.14
Indole-3-lactic acid	$[M-H]^-$	204.0666	3.67	204.0664	4.50	4.69	0.19
Ile-Pro	$[M+H]^+$	229.1546	2.39	229.1554	2.39	2.52	0.13
Thr-Leu	$[M+H]^+$	233.1503	2.04	233.1504	2.20	1.92	−0.28
Leu-Leu	$[M+H]^+$	245.1869	3.17	245.1874	3.81	3.84	0.03
Val-Phe	$[M+H]^+$	265.1571	2.89	265.1560	3.35	3.36	0.01
Oxo-octadecadienoic acid	$[M+H]^+$	295.2294	6.17	295.2284	9.04	8.93	−0.11
Chrysoeriol	$[M+H]^+$	301.0726	5.14	301.0719	6.91	7.18	0.27
12,13-DiHOME	$[M-H]^-$	315.2555	6.20	315.2543	9.07	8.99	−0.08
Phytosphingosine	$[M+H]^+$	318.3046	6.00	318.3025	8.82	8.66	−0.17
DIBOA-dihexoside	$[M-H]^-$	504.1335	2.92	504.1366	3.51	3.42	−0.09

Table S4. Other characteristics of the significantly correlating metabolites with *L. plantarum*, including universal identifiers, level of identification, and main observed MS/MS fragments. * Identification level according to the Metabolomics Standards Initiative recommendation: 1 = identified based on a reference standard, 2 = putatively identified based on spectral similarity to publicly available databases, 3 = putatively characterised compound class based on LC-MS properties, such as characteristic MS/MS fragments and retention time.

	Identifiers				
Curated ID	HMDB ID	PubChem CID	ID level*	Ion	Main MS/MS fragments: *m/z* (relative intensity)
α-Hydroxyisovaleric acid	HMDB0000407	99823	2	−	71.052 (100), 117.057 (58)
2-Hydroxyisocaproic acid	HMDB0000746	92779	2	−	85.066 (100), 131.069 (32), 69.036 (6), 113.057 (5)
Indole-3-carboxaldehyde	HMDB0029737	10256	2	−	144.045 (100), 116.050 (11)
N-(2-Hydroxyphenyl)-acetamide (HPAA)	HMDB0061919	11972	2	−	108.046 (100), 90.034 (10)
3-Phenyllactic acid	HMDB0000779	3848	2	−	147.044 (100), 103.056 (82), 165.057 (37), 119.046 (31), 72.994 (13), 101.035 (10), 91.053 (7)
Suberic acid	HMDB0000893	10457	1	−	111.081 (100), 173.084 (32), 83.049 (13), 57.033 (4)
2-Isopropylmalic acid	HMDB0000402	77	2	−	115.040 (100), 85.065 (98), 175.061 (66), 113.063 (47), 131.070 (24), 59.014 (12)
Caffeic acid	HMDB0001964	689043	1	−	135.046 (100), 179.037 (33), 44.999 (20)
Dihydrocaffeic acid	HMDB0000423	348154	1	−	137.061 (100), 135.047 (70), 43.001 (59), 93.002 (58), 59.012 (44)
4-Hydroxyphenyllactic acid	HMDB0000755	9378	2	−	163.039 (100), 135.045 (72), 72.994 (55), 119.049 (35), 181.053 (33), 74.000 (26), 134.035 (19), 93.037 (18)
Azelaic acid	HMDB0000784	2266	1	−	125.098 (100), 187.100 (50), 169.089 (22), 97.066 (20), 57.034 (7)
Ferulic acid	HMDB0000954	445858	1	−	134.038 (100), 178.030 (32), 149.063 (13), 193.054 (10), 117.036 (4)
Dihydroferulic acid	HMDB0062121	14340	2	−	136.052 (100), 121.028 (33), 44.999 (12), 135.046 (10)
Sebacic acid	HMDB0000792	5192	2	−	201.115 (100), 89.025 (75), 139.110 (62), 183.105 (62), 111.076 (13), 57.032 (12)
Indole-3-lactic acid	HMDB0000671	92904	2	−	158.060 (100), 204.067 (98), 142.065 (38), 116.050 (37), 186.061 (24), 72.993 (22)
Ile-Pro	HMDB0011174	444876	2	+	116.071 (100), 86.098 (61), 70.065 (38), 229.168 (30), 81.075 (16), 88.956 (14)
Thr-Leu	HMDB0029065	7021828	2	+	74.0627 (100), 187.150 (16), 233.154 (12), 56.049 (12), 86.099 (10), 132.101 (10)
Leu-Leu	HMDB0028933	76807	2	+	44.050 (100), 86.098 (80)
Val-Phe	HMDB0029134	6993120	2	+	55.053 (100), 72.080 (72)
Oxo-octadecadienoic acid	–	–	3	+	55.054 (100), 67.054 (54), 79.053 (29), 93.070 (25), 43.054 (20), 107.085 (14)
Chrysoeriol	HMDB0030667	5280666	2	+	301.071 (100), 286.048 (6), 258.053 (2), 229.048 (1)
12,13-DiHOME	HMDB0004705	10236635	2	−	43.055 (100), 81.071 (88), 79.056 (55), 67.055 (50), 93.069 (38), 55.054 (32)
Phytosphingosine	HMDB0004610	122121	2	+	318.300 (100), 60.046 (62), 300.288 (18), 282.280 (15), 270.282 (4)
DIBOA-dihexoside	n/a	139292137	2	−	134.025 (100), 162.020 (57), 89.025 (11)
